# Reframing dementia care in the era of disease-modifying therapies: informational, psychosocial, and systemic insights from Japan

**DOI:** 10.1186/s12913-026-14472-8

**Published:** 2026-03-30

**Authors:** Kae Ito, Shuji Tsuda, Taisei Wake, Akira Hatakeyama, Fumiko Ogisawa, Mayuko Ono, Riko Nakayama, Tomoko Wakui, Nobuko Nagano, Atsushi Iwata

**Affiliations:** 1 Tokyo Metropolitan Institute for Geriatrics and Gerontology, 35-2 Sakae-cho Itabashi-ku, Tokyo, 173-0015 Japan; 2 Department of Psychology and Social Welfare, Seigakuin University, 1-1 Tosaki, Ageo-shi, Saitama, 362-8585 Japan

**Keywords:** Dementia, Disease-modifying therapy, Care pathways, Post-diagnostic support, Psychosocial support, Service coordination

## Abstract

**Background:**

The introduction of disease-modifying therapies (DMTs) for Alzheimer’s disease has prompted major changes in diagnostic pathways, referral processes, and service coordination in dementia care. Japan, as an early adopter of DMTs within a nationally coordinated dementia-care framework, offers an opportunity to examine how health service structures respond to these changes. This study explored health service–related support needs emerging across the DMT pathway, focusing on patient and informal caregiver experiences, with complementary perspectives from service providers.

**Methods:**

A qualitative study was conducted using semi-structured interviews with 48 participants, including nine patients who underwent DMT eligibility assessment, seven informal caregivers, 11 physicians, four nurses, five clinical psychologists, five social workers, and seven community-based dementia support providers. Data were analyzed using the Framework Method, an applied qualitative approach suitable for health services research. Patients’ and caregivers’ accounts were treated as the primary analytic focus, while provider perspectives were used to contextualize system-level factors influencing care delivery.

**Results:**

Three interrelated themes were identified. First, informational support needs reflected inequitable access to trustworthy information, difficulties in sustaining understanding of complex medical explanations, and a lack of structured opportunities to revisit information over time, particularly during transitions such as ineligibility, treatment discontinuation, or completion. Second, psychosocial support needs were closely shaped by service processes, including stigma-related experiences across clinical and social contexts, family-related tensions around treatment decisions, fluctuating expectations regarding treatment effects, and limited support for adjustment when DMT was no longer an option. Third, systemic and collaborative support needs highlighted fragmented roles between primary care and DMT-designated institutions, unclear referral and handover pathways, insufficient psychosocial care capacity, and weak integration between DMT delivery systems and existing dementia-care services.

**Conclusions:**

The implementation of DMTs has amplified pre-existing gaps in dementia care systems, revealing previously underrecognized structural vulnerabilities across informational, psychosocial, and systemic domains. Findings indicate that DMTs should be embedded within coordinated care pathways that ensure continuity of information provision, access to psychosocial support, and clear allocation of follow-up responsibility regardless of treatment eligibility. Aligning pharmacological innovation with health service design is essential to support equitable, continuous, and person-centered dementia care.

**Supplementary Information:**

The online version contains supplementary material available at 10.1186/s12913-026-14472-8.

## Background

The introduction of anti-amyloid beta disease-modifying therapies (DMTs) has altered the landscape of Alzheimer’s disease (AD) care, shifting clinical practice from purely symptomatic management toward interventions targeting the biological underpinnings of the disease [1, 2].

These therapies aim to slow cognitive and functional decline by reducing beta-amyloid accumulation in the brain and are currently indicated for individuals with mild cognitive impairment due to AD or early-stage AD. As AD accounts for 60–70% of cases of dementia in older persons [3], the implementation of DMTs has broad implications for dementia care. In addition, treatment requires regular intravenous infusions, typically administered every two to four weeks, along with ongoing monitoring for safety.

The implementation of these therapies can trigger system-wide changes—new diagnostic pathways, rapid assessment units, and the expansion of multidisciplinary teams to ensure the safe administration and monitoring [1]. Such structural adaptations are now required to deliver DMT safely and effectively in dementia care.

Alongside these structural changes, a substantial body of research has consistently demonstrated the importance of psychosocial support in enabling individuals to live well with dementia [4]. These approaches are often conceptualized within the framework of post-diagnostic support, which is commonly defined as holistic and continuous care that responds to the progressive nature of dementia and addresses the needs of both patients and informal caregivers [5]. However, service evaluations and systematic reviews consistently indicate that such support remains fragmented and insufficient to meet diverse and evolving needs [6–9]. Although earlier access to support is associated with better outcomes, a recent systematic review reported that people with dementia and their caregivers often seek assistance only when symptoms become severe [10].

By promoting earlier diagnosis and sustained engagement with healthcare systems, DMTs bring these longstanding gaps in post-diagnostic support into sharper focus, extending them into the early post-diagnostic and even pre-diagnostic stages. The introduction of DMTs therefore creates not only new therapeutic opportunities but also an imperative to reconsider what constitutes appropriate and timely post-diagnostic support for all persons living with dementia.

Japan provides an illustrative context in which to examine these challenges. The country has established a nationwide dementia care system centered on dementia-specialized institutions known as Medical Centers for Dementia (MCDs), which serve as regional hubs for diagnosis, multidisciplinary care, and post-diagnostic support. However, because many MCDs lack the infrastructure required for safe DMT administration, a separate framework for DMT delivery has been developed [11]. Recent observations have suggested that the implementation of a DMT delivery system in Japan, while designed to ensure safe and equitable access to treatment, may inadvertently contribute to discontinuities in post-diagnostic support, particularly during transitions between providers within the DMT care pathway [12]. This functional bifurcation between the existing dementia care system and the emerging DMT delivery pathway risks widening gaps between diagnosis and ongoing support, particularly for individuals who are deemed ineligible for DMT, discontinue treatment due to medical or practical reasons, or require ongoing support after treatment completion.

## Methods

### Aim

This study aimed to examine how the introduction of DMTs reshapes psychosocial support needs related to health services, as reflected in the experiences of patients and informal caregivers, across pre- and post-diagnostic phases.

### Data collection

A qualitative study using semi-structured interviews was conducted between May 9 and September 11, 2025. To understand the evolving support needs of patients and informal caregivers in the context of DMT implementation, we deliberately selected two groups for sampling: (1) primary participants, individuals who underwent DMT eligibility assessment (eligible, ineligible, or discontinued) and their informal caregivers; and (2) secondary participants, as contextual informants, including healthcare professionals at DMT-designated institutions and/or MCDs, as well as community-based dementia support providers, such as executives from family associations and staff of dementia support centers.

Patients and their informal caregivers were recruited from the DMT outpatient service at Hospital A, a designated DMT institution that also serves as a MCD, in an urban area of Japan. Hospital A served as the primary study site. Service providers were recruited using convenience and snowball sampling. They comprised professionals from multiple hospitals, clinics, and community settings across urban and rural regions of Japan involved in DMT assessment, administration, or care coordination. Recruitment continued until thematic sufficiency was achieved. Two patients and two informal caregivers declined participation; no reasons were requested or recorded. There were no dropouts after consent was obtained.

All participants received detailed written and verbal explanations regarding the study purpose and interview procedures, and written informed consent was obtained prior to participation. Interviews were conducted in Japanese in person (*n* = 44) or via Zoom (*n* = 4), lasting 30–60 min, by six trained qualitative researchers (KI, AH, FO, MO, RN, TW). The interviewers included five women and one man and comprised a multidisciplinary team consisting of a geriatric psychiatrist (MD, PhD), a social worker (with graduate-level training in dementia care research), two clinical psychologists (one PhD, one with graduate-level training in dementia care research), a nurse (PhD), and a researcher specializing in family caregiving of persons living with dementia (PhD). All interviewers had prior experience conducting qualitative interviews and were actively involved in dementia-related clinical or community practice at the time of the study. Face-to-face interviews were conducted at locations chosen by participants. For patients and informal caregivers, interviews were held either in private rooms within the hospital or at their homes, depending on their preference. Interviews with service providers were conducted at their workplaces (e.g., offices or clinics). Interviews conducted via Zoom were held at locations selected by participants, including hospitals, homes, or offices. The presence of additional persons during interviews was determined by participant preference. Some patients chose to have a family member present during the interview. No other individuals were present during interviews with service providers.

The semi-structured interview guides included open-ended questions designed to explore psychosocial experiences and perceived support needs arising from the introduction of DMT. They covered understanding of medical information, emotional responses to eligibility determination, decision-making, family dynamics, perceived treatment effects, post-assessment transitions, and care coordination. For details, see Supplementary File 1. Each participant was interviewed once; repeat interviews were not conducted. Interviews were audio-recorded, transcribed verbatim, and de-identified. Field notes were not systematically recorded. Transcripts were not returned to participants for comment or correction. Participants were informed that the interviewers were clinicians and researchers specializing in dementia care. Interviewers did not have prior personal or therapeutic relationships with patient participants or their informal caregivers before study commencement. Some service providers were professionally known to members of the research team through prior clinical or academic collaboration; however, interviews were conducted with an emphasis on maintaining neutrality and minimizing potential role-related influence.

### Data analysis

Data were analyzed using the Framework Method [13], which is well suited for applied health research involving multiple participant groups. Consistent with the five stages of framework analysis—data familiarization, framework identification, indexing, charting, and mapping and interpretation—analysis proceeded as follows. First, transcripts were read repeatedly to achieve familiarization with the data. Initial codes were generated inductively and discussed among the analytic team to develop a working analytical framework. Second, the framework was systematically applied to all transcripts (indexing). Data were then charted into matrices organized by participant group and phase of the care pathway to facilitate comparison across cases and themes. Primary participants (patients and informal caregivers) and secondary participants (service providers) were initially charted in separate matrices to preserve the integrity of each perspective. In the final stage (mapping and interpretation), patterns within and across matrices were examined to explore convergences, divergences, and relational dynamics. While patient–caregiver accounts constituted the primary analytic focus, provider perspectives were used to contextualize and interpret these experiences within broader service-level structures.

KI and ST independently coded transcripts and resolved discrepancies through discussion; themes were refined iteratively with input from KI, TW, and ST. Coding and data management were conducted using MAXQDA 2024 (VERBI Software, Berlin, Germany).

The analytic team comprised three researchers (a woman and two man), including a geriatric psychiatrist (MD, PhD), a geriatric physician (MD, PhD), and a clinical psychologist (PhD). All had experience in qualitative methodology and dementia care research. Interpretations were discussed among the researchers to ensure credibility and consistency of coding. Participants did not provide feedback on the findings.

Ethical approval was obtained from the Institutional Review Board and Ethics Committee of the Tokyo Metropolitan Institute for Geriatrics and Gerontology (Approval No. R24-121).

## Results

### Overview of the participants

A total of 48 participants took part in the study. Participant characteristics are summarized in Table 1. To protect participant anonymity, demographic characteristics are reported in aggregate form only, including age ranges (by decade) and overall gender distribution. Primary participants comprised nine individuals with Alzheimer’s disease who underwent DMT eligibility assessment and seven of their informal caregivers. Among patients, seven initiated DMT, including one who later discontinued, while two were considered ineligible. Patients included one person in their 60s, four in their 70s, four in their 80s; three were men and six were women. Among informal caregivers, five cared for individuals who initiated DMT, including one whose care recipient discontinued treatment, and two cared for individuals who did not receive DMT. Caregivers included two individuals in their 50s, two in their 60s, and three in their 70s; four were men and three were women.

**Table 1:** Participants’ characteristics

Attribute	No.			
Patient (Pt)	1	Ineligible patient		
	2	Ineligible patient		
	3	Eligible patient, discontinued		
	4	Eligible patient		
	5	Eligible patient		
	6	Eligible patient		
	7	Eligible patient		
	8	Eligible patient		
	9	Eligible patient		
				**Relationship with the patient**
Informal caregiver (IC)	1	IC of ineligible patient		Daughter of Pt 1
	2	IC of ineligible patient		Sister of Pt 2
	3	IC of eligible patient		Wife
	4	IC of eligible patient, discontinued		Husband of Pt 3
	5	IC of eligible patient		Husband
	6	IC of eligible patient		Son
	7	IC of eligible patient		Husband of Pt 5
		**Affiliation**	**Types of affiliation**	**Specialty**
Physician (Dr)	1	Hospital A	MCD, DMTi	Psychiatrist
	2	Hospital A	MCD, DMTi	Psychiatrist
	3	Hospital A	MCD, DMTi	Psychiatrist
	4	Hospital A	MCD, DMTi	Neurologist
	5	Hospital A	MCD, DMTi	Neurologist
	6	Hospital B	MCD, DMTi	Neurologist
	7	Hospital C	MCD, DMTi	Psychiatrist
	8	Hospital D	DMTi	Neurologist
	9	Clinic E	DMTi	General practitioner
	10	Clinic F	MCD	Psychiatrist
	11	Clinic G	MCD	Psychiatrist
Nurse (Ns)	1	Hospital A	MCD, DMTi	
	2	Hospital A	MCD, DMTi	
	3	Hospital H	MCD	
	4	Clinic E	DMT	
Clinical psychologist (CP)	1	Hospital A	MCD, DMTi	
	2	Hospital A	MCD, DMTi	
	3	Hospital A	MCD, DMTi	
	4	Hospital A	MCD, DMTi	
	5	Hospital H	MCD	
Social worker (SW)	1	Hospital A	MCD, DMTi	
	2	Hospital A	MCD, DMTi	
	3	Hospital I	MCD, DMTi	
	4	Hospital J	MCD	
	5	Clinic K	MCD, DMTi	
		**Affiliation**		**Position/Occupation**
Community-based dementia support providers (CS)	1	Family association for persons with dementia L		Executive
	2	Family association for persons with dementia M		Executive
	3	Family association for persons with dementia M		Executive
	4	Support Center for Early Onset Dementia N		Care worker
	5	Support Center for Early Onset Dementia N		Social worker
	6	Support Center for Early Onset Dementia N		Social worker
	7	Support Center for Early Onset Dementia N		Nurse

Abbreviation: MCD, Medical Center for Dementia; DMTi, disease-modifying therapies-designated institution

Secondary participants included 11 physicians, four nurses, five clinical psychologists, five social workers, and seven community-based dementia support providers involved in DMT delivery or post-diagnostic support.

### Pre- and post-diagnostic support needs of patients and informal caregivers involved in the DMT process

Thematic analysis revealed three main themes that represented support needs related to DMT implementation: (1) informational support needs; (2) emotional and psychosocial support needs; and (3) systemic and collaborative support needs.

In this study, informational support needs were treated as a distinct domain, capturing issues related to access to, comprehension of, and continuity of medical information. Although informational support is often conceptualized as part of broader psychosocial support, we distinguished it analytically due to its central role in shaping treatment eligibility, decision-making, and care navigation within the DMT pathway. Emotional and psychosocial support needs were defined as emotional and relational processes shaping how individuals manage DMT-related experiences, including stigma, interpersonal tensions, emotional changes, and meaning reconstruction. Systemic and collaborative support needs encompassed structural and organizational factors influencing coordination, role clarity, and continuity of care across providers within the DMT pathway.

Each theme comprised several subthemes derived from patient and informal caregiver accounts (primary data), with perspectives from service providers (secondary data) used to contextualize system-level factors. A structured overview of the themes and subthemes, including their operational definitions, is presented in Table 2.

**Table 2:** Framework matrix of pre- and post-diagnostic care needs related to disease-modifying therapy (DMT) implementation

				Patients & Informal Caregivers (Primary)	Service Providers (Secondary)
**Theme 1**	**Informational Support Needs**				
**Subtheme**	**1.1**	**The need for equitable and trustworthy access to information**			
	**Code**	**1.1a**	Prevalence of negative public discourse	*No direct verbatim statements from patients or caregivers were identified for this code.*	When patients are deemed ineligible, some of them criticize the treatment itself and even those who received it. (CS2) Ordinary people often react fearfully to terms like “brain edema,” saying that DMT sounds frightening. I think those comments can discourage patients from considering treatment. (CS1)
	**Code**	**1.1b**	Limited access to accurate and comprehensible information	I tried searching on Google, but I couldn’t figure anything out. Then a colleague told me where I should go. (Pt2) I don’t really search for information online, so I learned about DMT from a flyer for a public lecture. (Pt1) I learned about DMT from TV and decided to visit a hospital. (Pt5, IC7)	Necessary information does not reach those who need it. (Dr2) Younger people look up information online, but older persons do not. (Dr4) Online information alone cannot reach older persons. We need other media, including printed materials. (PSW1)
	**Code**	**1.1c**	Limited access to guidance about social and welfare consultation	I didn’t know where to go for advice and wished someone at the hospital had guided me. (Pt4) I don’t know anything or who to ask about the social welfare system or what kind of support I can get for our daily living. I wish someone at the hospital had told me where I could go for advice. (IC4)	Some people come to ask us (social workers) about things they can’t ask their physicians. (PSW1)
**Subtheme**	**1.2**	**The need for support in sustaining and reinforcing understanding of DMT risks**,** effects**,** and limitations**			
	**Code**	**1.2a**	Difficulties in achieving initial comprehension of DMT explanations	No matter how much the doctor explains, I just can’t understand it, so I have to rely on the doctor. (Pt4) My wife doesn’t understand what kind of medication it is, what effects it has, or what the treatment actually involves. (IC4)	Patients with very early MCI usually understand our explanations, but those with more cognitive decline struggle. Younger family members who search for information online before visiting the hospital tend to understand more easily. Older family members often have difficulty understanding our explanations. (Dr4) Some people worry too much about side effects, while others worry too little. Patients and families often take terms like ‘brain edema’ or ‘micro bleeds’ too literally—they know the words but do not understand what they actually mean. (Dr1) I felt that the patient did not fully understand the doctor’s explanations. The family members seemed to understand that DMT slows progression. Those who were familiar with medical procedures appeared to grasp both its effects and its limitations better. (CP2)
	**Code**	**1.2b**	Difficulties in retaining information and aligning understanding over time	I think the explanation was understandable at the time, but I don’t remember what the doctor told me. (Pt1、Pt5、Pt7) I don’t remember whether the doctor explained it to me or not. (Pt3)	*No direct verbatim statements from care providers were identified for this code.*
**Subtheme**	**1.3**	**The need for ongoing dialogue to recalibrate expectations and update understanding as circumstances change**			
	**Code**	**1.3a**	The need to recalibrate expectations and update understanding as treatment progresses	I’m not sure whether DMT is really effective, but I can’t ask because the doctor is so busy. (IC3) Television programs say that some people improve and others do not, so I try not to expect too much. (Pt6)	Even when patients and families seem to understand the effects and limitations of DMT, once therapy begins, they repeatedly ask ‘How many injections until dementia is cured?’ I think that’s because they want to believe that the treatment will cure dementia. (Dr5)
**Subtheme**	**1.4**	**The need for medical guidance during post-treatment transitions and follow-up care (ineligibility/ discontinuation/ completion)**			
	**Code**	**1.4a**	Uncertainty about post-DMT medical follow-up and clinical points of contact	What will happen to my mother after DMT ends? The treatment may end, but she will still have dementia. (IC6) We were just told to go back to the primary care physician, but he is not a dementia specialist. I wished there had been somewhere that could tell us what to do next. (IC4)	After a patient was deemed ineligible because his MCI was not considered to be due to AD, his family became anxious. They knew conversion was possible, but they didn’t know which medical facility to turn to for help. (CP2) Families often ask me what happens after completing DMT. (Dr5) Some patients need to revisit and relearn information about DMT. They need time to understand that they are not eligible, and to come to terms with it emotionally. (CP3)
**Theme 2**	**Emotional and Psychosocial Support Needs**				
**Subtheme**	**2.1**	**The need for support to address stigma-driven fear and ambivalence**			
	**Code**	**2.1a**	Exposure to public stigma	When we visited the hospital to see if my mother was eligible for DMT, the doctor said to her, “Do you really want to receive DMT even though you’re over 80?” I felt that was ageism. (IC6)	One patient confessed to me that she felt hurt because the doctor spoke mainly to her daughter instead of to her. She said she wanted the explanations to be given directly to her. (CP4) I don’t think there’s any point in people over 80 spending money on DMT. (CS2)
	**Code**	**2.1b**	Stigma-related fears and diagnosis-acceptance challenges	My father had dementia and was placed in a nursing home because of wandering. I have a strong fear that I might end up the same way, and this fear is my motivation to receive DMT. (Pt3)	Receiving treatment and accepting the diagnosis are different things. People may deny their memory problems and, at the same time, they are hoping to get better. I think it’s a very complicated state of mind. (CP1) Patients and families have different views of dementia. We have to be aware of these differences and try to reduce stigma when we engage with them. (PSW1)
**Subtheme**	**2.2**	**The need for support to navigate family-related emotional tensions**			
	**Code**	**2.2a**	Family-driven expectations and pressure toward the patient regarding DMT	I don’t feel I have a memory problem and I’m not particularly interested in DMT. But my husband/wife strongly encouraged me to start the treatment, and I didn’t feel I could refuse. (Pt5,7) Sometimes I feel like quitting because it doesn’t seem to be working, but when I think about how much my family is counting on me and all the effort they make to take me to appointments, I cannot bring myself to say so. (Pt9) My wife doesn’t think she needs treatment and resists going to the hospital every time. But I can’t just let her do as she likes when there is a treatment available. I feel it is my responsibility as her family. (IC4)	Families who strongly hope for improvement sometimes push patients toward DMT by emphasizing their cognitive decline, or continue seeking reassessment after a patient has been deemed ineligible. When I observe this, I feel it may damage patients’ sense of dignity. (Dr1) Families with high expectations sometimes ask patients to do cognitive training in addition to DMT. It seems to me that this places additional pressure and emotional strain on patients. (CP3) I think families with very strong expectations have their own anxiety, and that anxiety can become misdirected so that the treatment itself becomes the goal. (Dr2) When only family members are enthusiastic, the patient’s feelings may be overlooked. There was a case in which this led to the emergence of BPSD, making it difficult for the patient to continue DMT. (Dr2)
	**Code**	**2.2b**	Family role expectations and caregiving strain related to DMT	I feel sorry that my family has to take me to the hospital every two weeks. (Pt2, 8) When deciding whether my brother would receive DMT or not, the physician repeatedly told me that “it depends on the family” and that the family “needs to make an effort.” I felt compelled to take responsibility, and I sensed that people without family were implicitly excluded. (IC2) It was a burden. I had to take time off work on weekdays to make it work. (IC6) If I told my son/ daughter, he/she might feel obligated to come and check on me, even though he/she doesn’t live nearby. Maybe I’ll tell her when the disease progresses, not now. (Pt5, 6)	DMT requires substantial family involvement, which can be a strain. I therefore ask families, before obtaining informed consent, whether they can manage this. (Dr4) Patients and families seem to try not to burden each other. But because they do not communicate their true feelings, they often misread each other. And this creates strain in their relationship. (CP5)
**Subtheme**	**2.3**	**The need for support to manage expectations and emotional exhaustion related to DMT**			
	**Code**	**2.3a**	Difficulty perceiving treatment effects and resulting emotional distress	I’m not experiencing any problems now, so I can’t tell whether the infusion is working. (Pt6, IC5) I don’t feel any effect, and I even think the condition has worsened. My husband says it makes him sad. (IC7)	Patients seem to feel torn. They have expectations for DMT, but they also feel the physical and psychological strain of treatment. When they don’t see any clear effects, they begin to doubt whether they should continue. (Ns3) I’ve noticed that anxiety increases when patients or family members who were initially enthusiastic do not sense any clear effect. (Dr9)
	**Code**	**2.3b**	Reconfiguring family relationships and emotional responses during DMT	I enjoy thinking about where to have lunch after visiting the hospital for infusion with my husband. (Pt4) After being diagnosed with dementia and beginning DMT, our child started visiting more often out of concern, helping me share the caregiving I had been doing alone. Our grandchildren also began to visit, and I feel our family relationships have become gentler. (Pt3, IC5)	Among families who were initially enthusiastic, I sometimes see strong expectations that later turn into disappointment when no noticeable effects are perceived. Some become indifferent for a time, but eventually—after about a year—many settle into a more accepting view, saying the patient now seems “age-appropriate.” (CP4)
**Subtheme**	**2.4**	**The need for support in meaning reconstruction and acceptance when DMT is no longer an option**			
	**Code**	**2.4a**	Turning toward non-pharmacological and social support	I was disappointed to hear that I was not eligible for DMT. But the staff at the Community General Support Center regularly told me that there is a lot to do other than medication, and called me about local events. This helped me shift my mindset and actually became a positive turning point in how I live my life. (Pt1) I was disappointed that DMT wasn’t an option for my brother, but the staff at the support center helped us shift our mindset away from medication. We were able to face dementia head-on, which led us to build our own support networks and attend community gatherings. Having some routines or events gives his life more structure and energy. (IC2) Having lived with dementia, I do think the medication matters, but the depth of everyday relationships is just as important—family, friends, and people in the neighborhood. (IC5)	Although patients are often disappointed when they are deemed ineligible for DMT, I think most are eventually able to move forward. We explain there are still many things we can do together, even if DMT is not an option. We continue to offer psychosocial support, but they often refuse it at first. (Dr1) When patients are deemed ineligible for DMT, some of them criticize both the treatment itself and those who received it. I think they are just so envious of those who can receive the treatment. I think they are the ones who really need support. (CS2) When DMT is discontinued or terminated, I think peer support and psychoeducation can be helpful for patients who have psychosocial needs. (CP3)
**Theme 3**	**Systemic and Collaborative Support Needs**				
**Subtheme**	**3.1**	**The need for clarity regarding primary care physicians’ roles in DMT eligibility assessment and post-diagnostic support**			
	**Code**	**3.1a**	Inconsistencies in DMT eligibility evaluations between primary care physicians and DMT-designated institutions	My primary care physician recommended DMT, so I expected it to be a promising new treatment. I was shocked and disappointed to learn I wasn’t eligible. Why couldn’t my primary care physician evaluate properly? (Pt2)	Some primary care physicians lack the skills needed to appropriately assess very early-stage dementia or mild cognitive impairment. (CS4) There is also limited understanding that these conditions are now targets of treatment, and I think this is becoming a barrier to DMT implementation. (Dr1) I advise primary care physicians to confirm that there are no MRI contraindications or prior microbleeds, and to explain that DMT is administered by intravenous infusion before referring patients to us (DMT-designated institutions). But the situation is different for the MMSE. The MMSE scores can vary by a few points depending on the method and version used, so I believe dementia staging should be assessed by trained staff at DMT-designated institutions, not primary care physicians. (Dr5)
	**Code**	**3.1b**	Ambiguity in post-diagnostic support roles between primary care physicians and DMT-designated facilities	I would consult my primary care physician about everything, because he knows me best. (Pt1, Pt7) I don’t talk to my primary care physician about dementia because I’m already seeing a dementia specialist hospital for DMT. (Pt3) My primary care physician is so busy that I hesitate to consult him. (IC6) I’m not sure what I can talk about with the DMT doctor besides the infusion. (Pt5, Pt7)	After DMT starts, primary care physicians often leave dementia care, including post-diagnostic care, to the DMT facility, which makes me feel uncomfortable. (Dr6, SW4) For about eighteen months, patients receive care from both their primary care physician and the DMT-designated facility. But if they already have a primary care doctor, providing post-diagnostic support can create conflict, so we usually do not offer it. (Dr9)
**Subtheme**	**3.2**	**The need for coordinated post-DMT handover and clearly assigned follow-up responsibility**			
	**Code**	**3.2a**	Unclear post-DMT referral pathways causing confusion	When they told us that the DMT can’t be continued and that our visits to the clinic would also end, I felt as if we were being abandoned. (IC4) Where should we go after DMT ends? The physician told us that our primary care physician will take over, but he is not a specialist. (IC6)	When patients are deemed ineligible or complete DMT, they are usually redirected to their primary care physicians. But if they don’t have one, they are often not referred anywhere. (CP2) Some patients lose their connection with any medical institution after being deemed ineligible. They try to search for help on their own, and some eventually come to our clinic. (SW5) There is very little coordination across institutions, so patients often do not know where to go after DMT completion or discontinuation. (CS4) I think the local government needs to establish a more formal referral pathway. This is difficult to address at the level of individual institution. (Dr5)
	**Code**	**3.2b**	Delay in accessing post-diagnostic support	*No direct verbatim statements from patients or caregivers were identified for this code.*	I feel that because responsibility for post-diagnostic support is not clearly assigned, the system ends up prioritizing pharmacological treatment. As a result, non-pharmacological interventions often get delayed. (Dr2) I feel that for patients who are eligible for DMT, non-pharmacological interventions often get delayed. (CS4)
**Subtheme**	**3.3**	**The need for multidisciplinary collaboration to provide post-diagnostic support**			
	**Code**	**3.3a**	Insufficient psychosocial care capacity across primary care and DMT-designated institutions	It was just by chance that one of my brother’s colleagues introduced him to community dementia supporting services. Hospital staff is so busy that I don’t think they can provide that kind of support. (IC2)	Primary care physicians usually do not hire social workers or psychologists for their clinics. It seems to me that it is unrealistic to expect that they would provide post-diagnostic support. (Dr3, Dr4) At DMT-designated institutions outside of MCDs, like ours, we don’t have social workers or psychologists available for DMT patients. So I end up managing all support needs myself, which is impossible. (Dr8)
**Subtheme**	**3.4**	**Toward an integrated dementia care system that includes DMT**			
	**Code**	**3.4a**	Fragmented linkage between DMT-designated institutions and MCDs	*No direct verbatim statements from patients or caregivers were identified for this code.*	Post-diagnostic support is usually provided within MCDs and psychiatric services, while DMT is mainly delivered by neurologists and neurosurgeons. Sometimes they don’t even know what post-diagnostic support involves. (PSW5) Many DMT-designated institutions consider their role complete once DMT ends. In my opinion, referring patients to MCDs is appropriate and necessary. Yet this is currently not being done. (PSW3) I think effective collaboration between DMT-designated institutions and MCDs is ideal for providing post-diagnostic support, including support after DMT discontinuation and for those deemed ineligible. If that kind of collaboration existed, patients could continue receiving appropriate medical and social support as dementia progresses. (Dr4)
	**Code**	**3.4b**	DMT being positioned too narrowly within the broader continuum of dementia care	*No direct verbatim statements from patients or caregivers were identified for this code.*	If patients view anything other than DMT as an “inferior treatment,” they may be reluctant to consider other therapies or non-pharmacological approaches. I think we need to send a more positive message that options beyond DMT are also valuable. (Dr1) I don’t think DMT should be treated as something exceptional. Regardless of eligibility, patients should still have access to other medical and psychosocial support. (Dr1) If we place too much emphasis on DMT, we may end up in a situation where those who seek it become marginalized from the existing dementia care system. (PSW5)

Abbreviations: DMT; disease modifying therapy, MCD; Medical Center for Dementia, Pt; patient, IC; informal caregiver, Dr; physician, Ns; nurse, CP; clinical psychologist, PSW; psychiatric social worker, CS: community-based dementia support providers

In the thematic analysis, multiple codes and subthemes were often derived from individual participants’ accounts; plural expressions are used to describe analytically identified patterns rather than to indicate frequency.

## Theme 1: Information support needs

Across interviews, patients and informal caregivers described a range of informational support needs related to DMT across pre- and post-diagnostic phases. These included the need for equitable and trustworthy access to information, assistance in understanding and retaining complex explanations, opportunities to update information as circumstances evolved, and clear guidance following ineligibility, discontinuation, or completion of treatment.

### Subtheme 1.1 The need for equitable and trustworthy access to information

This subtheme captures both experiential and structural dimensions of unequal access to DMT-related information. Primary accounts described difficulties accessing accurate and comprehensible information and navigating available resources, whereas secondary data highlighted how public discourse and communication channels influence the circulation and reach of DMT-related information.

#### Code 1.1a Prevalence of negative public discourse

This topic emerged mainly through observations by service providers rather than patients’ spontaneous narratives. Service providers observed how public criticism and alarming terminology in non-medical contexts shaped perceptions of DMT. Such negative public discourse was described as contributing to fear and hesitation toward treatment.

*Ordinary people often react fearfully to terms like “brain edema*,*” saying that DMT sounds frightening. I think those comments can discourage patients from considering treatment. (CS1)*

This observation suggests that broader social narratives surrounding DMT may shape early care-seeking decisions.

#### Code 1.1b Limited access to accurate and comprehensible information

Primary data showed patients and caregivers, particularly older participants, relied primarily on non-digital sources such as television and printed materials for DMT-related information. Several patients and informal caregivers, including those in their 60s to 80s, described difficulties navigating online information.

*I tried searching on Google*,* but I couldn’t figure anything out. Then a colleague told me where I should go. (Pt2)*

This account illustrates how some older adults struggled to search for and locate relevant information online. Secondary data reinforced this pattern, highlighting generational differences in digital engagement. While younger individuals were generally able to navigate online information effectively, older persons often were not.

*Younger people look up information online*,* but older persons do not. (Dr4)*

Service providers acknowledged that system-level communication often relied on printed or web-based materials, which are intended to enable broad and timely dissemination of information. However, these channels may not be equally accessible to all older persons. As a consequence, those who might be candidates for DMT may not have access to appropriate information.

*Necessary information does not reach those who need it. (Dr2)*

These findings suggest that informational inequity arises not only from generational differences in digital engagement but also from system-level communication strategies that may fail to ensure equitable access.

#### Code 1.1c Limited access to guidance about social and welfare consultation

Primary data showed unmet needs for navigational information, including where to seek advice on social welfare and daily living support.

*I don’t know anything or who to ask about the social welfare system or what kind of support I can get for our daily living. I wish someone at the hospital had told me where I could go for advice. (IC4)*

Secondary data showed that individuals were reluctant to raise such concerns with physicians and often consulted social workers instead.

*Some people come to ask social workers about things they can’t ask their physicians. (PSW1)*

These narratives suggest that beyond medical decision-making, patients and caregivers experience uncertainty in navigating social welfare systems, pointing to a structural gap between clinical information provision and broader life-related guidance.

Taken together, Subtheme 1.1 indicates that informational inequity in the DMT pathway operates across interconnected levels. Primary accounts reveal difficulties in accessing accurate and comprehensible information and navigating both medical and social support systems. Secondary perspectives further illuminate how broader public discourse and institutional communication structures shape the availability and reach of information. These findings illustrate that informational access is shaped not only by individual-level experiences but also by structural conditions that influence how and to whom information becomes available.

### Subtheme 1.2 The need for support in sustaining and reinforcing understanding of DMT risks, effects, and limitations

This subtheme captures the challenges patients and caregivers faced not only in initially understanding explanations about DMT, but also in sustaining and recalibrating that understanding over time.

#### Code 1.2a Difficulties in achieving initial comprehension of DMT explanations

Primary data indicated difficulties in patients’ understanding of explanations about DMT risks, benefits, and treatment processes.

*No matter how much the doctor explains*,* I just can’t understand it*,* so I have to rely on the doctor. (Pt4)*

*My wife doesn’t understand what kind of medication it is*,* what effects it has*,* or what the treatment actually involves. (IC4)*

Secondary data similarly indicated substantial variation in comprehension depending on patients’ cognitive capacity and caregivers’ background knowledge.

*Patients with very early MCI usually understand our explanations*,* but those with more cognitive decline struggle. Younger family members who search for information online before visiting the hospital tend to understand more easily. Older family members often have difficulty understanding our explanations. (Dr4)*

Service providers also suggested that explanations were sometimes only partially retained or interpreted too literally.

*Some people worry too much about side effects*,* while others worry too little. Patients and families often take terms like ‘brain edema’ or ‘micro bleeds’ too literally—they know the words but do not understand what they actually mean. (Dr1)*

These accounts suggest that comprehension is shaped by multiple interacting factors, including cognitive capacity, knowledge, and how medical terminology was explained and contextualized during clinical encounters.

#### Code 1.2b Difficulties in retaining information and aligning understanding over time

Primary data showed that difficulties were expressed not only in understanding explanations but also in recalling them over time, even among participants who initially appeared to have understood the information.

*I think the explanation was understandable at the time*,* but I don’t remember what the doctor told me. (Pt1*,* Pt5*,* Pt7)*

*I don’t remember whether the doctor explained it to me or not. (Pt3)*

These accounts illustrate that even when explanations are initially understood, retention and integration of complex medical information may dimmish over time, particularly in the context of cognitive decline.

Taken together, Subtheme 1.2 illustrates that understanding DMT unfolds as a multi-stage process influenced by cognitive capacity, prior knowledge, temporal decline in information retention, and how medical information is explained and contextualized during clinical encounters. Primary narratives describe difficulties in both comprehension and retention of complex explanations, while secondary perspectives highlight how cognitive capacity and prior knowledge shape interpretation. Together, these accounts highlight how understanding requires repeated dialogue and reinforcement over time, particularly in light of differences in cognitive function and prior access to information.

### Subtheme 1.3 The need for ongoing dialogue to recalibrate expectations and update understanding as circumstances change

This subtheme captures the dynamic and evolving nature of expectations surrounding DMT and illustrates how understanding requires ongoing recalibration as treatment unfolds.

#### Code 1.3a The need to recalibrate expectations and update understanding as treatment progresses

Primary data indicated that participants expressed ongoing uncertainty about treatment effects, along with a desire to revisit explanations as treatment progressed.

*I’m not sure whether DMT is really effective*,* but I can’t ask because the doctor is so busy. (IC3)*

Others described attempting to manage expectations based on fragmented information from media sources.

*Television programs say that some people improve and others do not*,* so I try not to expect too much. (Pt6)*

These accounts illustrate how expectations were shaped not only by clinical explanations but also by fragmented media narratives, requiring patients to actively manage uncertainty.

Secondary data indicated that, although patients and families often appeared to understand the non-curative nature of DMT, questions about when dementia might be “cured” frequently re-emerged after treatment initiation. One provider interpreted this as reflecting a desire to believe in the possibility of cure.

*Even when patients and families seem to understand the effects and limitations of DMT*,* once therapy begins*,* they repeatedly ask ‘How many injections until dementia is cured?*’ *I think that’s because they want to believe that the treatment will cure dementia.* (*Dr5)*

Taken together, Subtheme 1.3 indicates that expectations about DMT evolve along the treatment trajectory rather than remaining fixed at the point of initial explanation. Primary accounts reflect ongoing uncertainty and hesitancy in raising questions, while secondary perspectives suggest that questions oriented toward cure and shifting expectations may re-emerge after treatment initiation. These findings illustrate how expectations require ongoing dialogue and recalibration over time, particularly as treatment experiences accumulate.

### Subtheme 1.4 The need for medical guidance during post-treatment transitions and follow-up care (ineligibility/ discontinuation/ completion)

This subtheme captures uncertainty surrounding continuity of medical care following DMT-related transitions, including ineligibility, discontinuation, or treatment completion.

#### Code 1.4a Uncertainty about post-DMT medical follow-up and clinical points of contact

Primary data indicated that participants expressed uncertainty about what medical care would follow DMT.

*What will happen to my mother after DMT ends? The treatment may end*,* but she will still have dementia. (IC6)*

Concerns about returning to a primary care physician who was “not a dementia specialist” were also described.

*We were just told to go back to the primary care physician*,* but he is not a dementia specialist. I wished there had been somewhere that could tell us what to do next. (IC4)*

Secondary data similarly showed that patients and informal caregivers often struggled to identify appropriate points of medical contact after ineligibility, discontinuation, or completion of DMT.

*After a patient was deemed ineligible because his MCI was not considered to be due to AD*,* his family became anxious. They knew conversion was possible*,* but they didn’t know which medical facility to turn to for help. (CP2)*

*Families often ask me what happens after completing DMT. (Dr5)*

It was also noted that among those who were deemed ineligible, some required additional informational support to process the situation. These accounts illustrate that transitions out of the DMT pathway were experienced not merely as clinical decisions but as moments of uncertainty requiring both informational and emotional processing.

*Some patients need to revisit and relearn information about DMT. They need time to understand that they are not eligible*,* and to come to terms with it emotionally. (CP3)*

Taken together, Subtheme 1.4 indicates that informational needs surrounding DMT extend beyond treatment eligibility. Primary narratives express uncertainty and concern about follow-up care, while secondary perspectives reveal systemic ambiguity regarding referral pathways and points of contact. Together, these findings illustrate how ambiguity surrounding referral pathways and points of contact contributes to uncertainty during post-DMT transitions.

Across Subthemes 1.1–1.4, a consistent pattern emerges: DMT-related informational support needs extend across the entire treatment trajectory rather than being confined to a single point of decision-making. Informational access, comprehension, expectation recalibration, and post-treatment transitions were experienced as interconnected processes unfolding over time. These findings illustrate how DMT-related support unfolds as a longitudinal and multi-stage process shaped by both individual cognitive factors and systemic communication structures.

## Theme 2: Emotional and psychosocial support needs

Whereas Theme 1 highlights informational gaps, Theme 2 captures emotional and psychosocial support needs that emerged along the DMT pathway. These needs included stigma-related experiences, family dynamics, emotional responses to treatment expectations, and adjustment when DMT was no longer an option.

### Subtheme 2.1 The need for support to address stigma-driven fear and ambivalence

This subtheme captures how stigma—both publicly expressed and internally experienced—shaped emotional responses to DMT eligibility, diagnosis, and treatment decisions.

#### Code 2.1a Exposure to public stigma

Primary data indicated that participants experienced stigma during clinical encounters, including feeling judged when treatment intentions were questioned based on age or frailty.

*When we visited the hospital to see if my mother was eligible for DMT*,* the doctor said to her*,* “Do you really want to receive DMT even though you’re over 80?” I felt that was ageism. (IC6)*

Secondary data similarly showed that service providers noticed instances in which communication bypassed patients and was directed toward family members, contributing to patients’ feelings of marginalization.

*One patient confessed to me that she felt hurt because the doctor spoke mainly to her daughter instead of to her. She said she wanted the explanations to be given directly to her. (CP4)*

Age-related stigma was also evident among the providers in community settings.

*I don’t think there’s any point in people over 80 spending money on DMT. (CS2)*

Even when framed as advice, such comments were described as being experienced as ageism by patients and families.

#### Code 2.1b Stigma-related fears and diagnosis-acceptance challenges

Primary data indicated that participants expressed fear shaped by internalized images of dementia. These fears were described as simultaneously motivating the pursuit of DMT while also generating anxiety about “becoming like that” if treatment proved ineffective.

*My father had dementia and was placed in a nursing home because of wandering. I have a strong fear that I might end up the same way*,* and this fear is my motivation to receive DMT. (Pt3)*

Secondary data further indicated that accepting a diagnosis and initiating treatment were experienced as psychologically distinct processes. Service providers also described instances in which patients appeared to minimize or deny symptoms while simultaneously hoping for improvement.

*Receiving treatment and accepting the diagnosis are different things. People may deny their memory problems and*,* at the same time*,* they are hoping to get better. I think it’s a very complicated state of mind. (CP1)*

Taken together, Subtheme 2.1 indicates that stigma influences emotional responses to DMT in both externally expressed and internally experienced forms. Primary narratives describe feeling judged in clinical contexts alongside internalized fear and ambivalence toward diagnosis and treatment. Secondary perspectives illustrate how communication practices and societal attitudes interact with these emotional responses. Together, these findings illustrate how decision-making about DMT are embedded within broader social meanings attached to dementia and later life.

### Subtheme 2.2 The need for support to navigate family-related emotional tensions

This subtheme captures the complex emotional dynamics experienced by patients and family members, who are often assumed to be the primary caregivers when considering and undergoing DMT. These dynamics include pressure related to social obligation, role expectations, and caregiving strain.

#### Code 2.2a Family-driven expectations and pressure toward the patient regarding DMT

Primary data indicated that differences in perceptions of DMT between patients and their family members often generated emotional tension.

*I don’t feel that I have a memory problem and I’m not particularly interested in DMT. But my husband/wife strongly encouraged me to start the treatment*,* and I didn’t feel I could refuse. (Pt5*,*7)*

Patients expressed difficulty articulating ambivalence toward receiving DMT.

*Sometimes I feel like quitting because it doesn’t seem to be working*,* but when I think about how much my family is counting on me and all the effort they make to take me to appointments*,* I cannot bring myself to say so. (Pt9)*

Family members who serve as informal caregivers described a sense of obligation to pursue treatment.

*My wife doesn’t think she needs treatment and resists going to the hospital every time. But I can’t just let her do as she likes when there is a treatment available. I feel it is my responsibility as her family. (IC4)*

These accounts illustrate how family members’ sense of responsibility may translate into pressure on patients to pursue or continue DMT.

Secondary data showed that service providers observed situations in which strong family expectations placed pressure on patients.

*Families who strongly hope for improvement sometimes push patients toward DMT by emphasizing their cognitive decline*,* or continue seeking reassessment after a patient has been deemed ineligible. When I observe this*,* I feel it may damage patients’ sense of dignity. (Dr1)*

*Families with high expectations sometimes ask patients to do cognitive training in addition to DMT. It seems to me that this places additional pressure and emotional strain on patients. (CP3)*

Providers also noted that when family members were highly invested in treatment outcomes, patients’ own emotional responses could be overlooked.

*When only family members are enthusiastic*,* the patient’s feelings may be overlooked. There was a case in which this led to the emergence of BPSD*,* making it difficult for the patient to continue DMT. (Dr2)*

These accounts illustrate how strong family expectations surrounding DMT, together with an implicit sense of obligation to pursue available treatment, can generate emotional pressure within family relationships. In this context, patients may find it difficult to articulate ambivalence or hesitation, while family members themselves navigate feelings of responsibility and social expectation.

#### Code 2.2b Family role expectations and caregiving strain related to DMT

Primary data indicated that participants recognized that receiving DMT required substantial family involvement. Family caregivers described taking on these roles despite feeling burdened.

*When deciding whether my brother would receive DMT or not*,* the physician repeatedly told me that “it depends on the family” and that the family “needs to make an effort.” I felt compelled to take responsibility*,* and I sensed that people without family were implicitly excluded. (IC2)*

*It was a burden. I had to take time off work on weekdays to make it work. (IC6)*

Patients expressed feelings of guilt, self-restraint, and hesitation about relying on family support, sometimes delaying disclosure to avoid “burdening” relatives.

*I feel sorry that my family has to take me to the hospital every two weeks. (Pt2*,* 8)*

*If I told my son/ daughter*,* he/she might feel obligated to come and check on me*,* even though he/she doesn’t live nearby. Maybe I’ll tell her when the disease progresses*,* not now. (Pt5*,* 6)*

Secondary data further indicated that service providers were aware of the substantial family involvement required for DMT and its potential strain.

*DMT requires substantial family involvement*,* which can be a strain. I therefore ask families*,* before obtaining informed consent*,* whether they can manage this. (Dr4)*

Service providers also described miscommunication within families.

*Patients and families try not to burden each other*,* but because they do not communicate their true feelings*,* they often misread each other*,* creating strain in their relationship. (CP5)*

These accounts illustrate how anticipated burden within family relationships shaped patients’ emotional responses, decisions about disclosure, and patterns of care-seeking.

Taken together, Subtheme 2.2 suggests that DMT-related decisions unfold within complex family relationships in which expectations, responsibility, and caregiving demands interact. Family members’ hope and sense of obligation may generate pressure on patients to initiate or continue treatment, sometimes making it difficult to express ambivalence. At the same time, the substantial practical involvement required for DMT can reorganize family roles, increase caregiving strain, and contribute to patterns of silence or emotional self-restraint within families. These dynamics illustrate how treatment-related decisions are shaped not only by medical considerations but also by relational expectations and perceived burdens embedded within family life.

### Subtheme 2.3 The need for support to manage expectations and emotional exhaustion related to DMT

This subtheme captures the emotional consequences of prolonged treatment engagement, particularly when expectations regarding effectiveness were uncertain or unmet.

#### Code 2.3a Difficulty perceiving treatment effects and resulting emotional distress

Primary data indicated that participants experienced difficulties in perceiving treatment effects.

*I’m not experiencing any problems now*,* so I can’t tell whether the infusion is working. (Pt6*,* IC5)*

*I don’t feel any effect*,* and I even think the condition has worsened. My husband says it makes him sad. (IC7)*

Secondary data highlighted the tension between initial hope and later uncertainty.

*Patients seem to feel torn. They have expectations for DMT*,* but they also feel the physical and psychological strain of treatment. When they don’t see any clear effects*,* they begin to doubt whether they should continue. (Ns3)*

Service providers also described increased anxiety when clear benefits were not perceived.

*I’ve noticed that anxiety increases when patients or family members who were initially enthusiastic do not sense any clear effect. (Dr9)*

These findings suggest that when perceived benefits remain unclear, hope may gradually shift toward doubt, generating emotional strain for both patients and families.

#### Code 2.3b Reconfiguring family relationships and emotional responses during DMT

Primary data indicated that participants described adjustments in family relationships following the introduction of DMT. Shared hospital visits were sometimes described as opportunities to spend time together.

*I enjoy thinking about where to have lunch after visiting the hospital for infusion with my husband. (Pt4)*

Others noted that introduction of DMT worked as a chance which family members who did not live with the patient became more involved.

*After being diagnosed with dementia and beginning DMT*,* our child started visiting more often out of concern*,* helping me share the caregiving I had been doing alone. Our grandchildren also began to visit*,* and I feel our family relationships have become gentler. (Pt3*,* IC5)*

These adjustments were described alongside the emergence of gentler or more supportive family interactions.

Secondary data showed that, although disappointment related to the lack of noticeable treatment effects was common initially, many families gradually recalibrated their expectations over time and developed a more accepting view of the patient’s condition.

*Among families who were initially enthusiastic*,* I sometimes see strong expectations that later turn into disappointment when no noticeable effects are perceived. Some become indifferent for a time*,* but eventually—after about a year—many settle into a more accepting view*,* saying the patient now seems “age-appropriate.” (CP4)*.

This shift from treatment-oriented expectations to age-based acceptance reflects a recalibration of meaning over time.

These findings suggest that DMT functioned not only as a medical intervention but also as a relational event that reconfigured family roles and emotional responses over time.

Taken together, Subtheme 2.3 indicates that uncertainty regarding treatment effects gives rise to evolving emotional and relational responses over time. While unclear or unmet expectations may generate fatigue, disappointment, and doubt, prolonged engagement with DMT can also prompt shifts in meaning and relational adjustment within families. These findings illustrate how emotional experiences related to DMT are not static but unfold dynamically, as hope, uncertainty, and acceptance are renegotiated within changing family contexts.

### Subtheme 2.4 The need for support in meaning reconstruction and acceptance when DMT is no longer an option

This subtheme captures how patients and families renegotiated meaning when DMT was unavailable due to ineligibility or discontinuation. Emotional responses often shifted from disappointment to gradual acceptance through engagement with non-pharmacological and social supports.

#### Code 2.4a Turning toward non-pharmacological and social support

Across cases of ineligibility, discontinuation, or treatment termination, similar psychosocial responses patterns were identified when support needs emerged. Primary data indicated that participants often experienced initial disappointment after being deemed ineligible for DMT, followed by a gradual shift toward non-pharmacological and community-based forms of support.

*I was disappointed to hear that I was not eligible for DMT. But the staff at the Community General Support Center regularly told me that there is a lot to do other than medication*,* and called me about local events. This helped me shift my mindset and actually became a positive turning point in how I live my life. (Pt1)*

Engagement with local services or community activities was described as helping reorient daily life and foster a sense of structure or connection.

*I was disappointed that DMT wasn’t an option for my brother*,* but the staff at the support center helped us shift our mindset away from medication. We were able to face dementia head-on*,* which led us to build our own support networks and attend community gatherings. Having some routines or events gives his life more structure and energy. (IC2)*

*Having lived with dementia*,* I do think the medication matters*,* but the depth of everyday relationships is just as important—family*,* friends*,* and people in the neighborhood. (IC5)*

Secondary data showed that psychosocial support was often declined initially, particularly soon after ineligibility decisions. Over time, however, patients and families were reported to engage with such support and to derive perceived benefit.

*Although patients are often disappointed when they are deemed ineligible for DMT*,* I think most are eventually able to move forward. We explain there are still many things we can do together*,* even if DMT is not an option. We continue to offer psychosocial support*,* but they often refuse it at first. (Dr1)*

At the same time, strong emotional reactions and substantial unmet support needs were also noted among individuals who were ineligible for DMT.

*When patients are deemed ineligible for DMT*,* some of them criticize both the treatment itself and those who received it. I think they are just so envious of those who can receive the treatment. I think they are the ones who really need support. (CS2)*

This response was interpreted by service providers as reflecting unresolved grief or perceived exclusion.

These accounts illustrate how ineligibility or discontinuation of DMT triggered a process of emotional recalibration, in which initial disappointment and resistance could gradually shift toward alternative forms of engagement and meaning-making.

Taken together, Subtheme 2.4 suggests that when DMT is no longer available, patients and families enter a process of emotional and relational recalibration rather than a single moment of acceptance. Initial disappointment may give way to gradual meaning reconstruction through engagement with non-pharmacological and community-based support. Primary narratives describe shifts in daily routines and relational priorities, while secondary perspectives highlight the importance of sustained encouragement even when support is initially declined. These findings suggest that adjustment following DMT ineligibility or discontinuation may unfold over time and can involve both emotional struggle and gradual adaptation.

Across Subthemes 2.1–2.4, a common pattern can be observed: emotional and psychosocial responses to DMT appear to unfold within relational and temporal contexts rather than at isolated moments of decision-making. Experiences of stigma, family-driven expectations, uncertainty about treatment effects, and adjustment following ineligibility were interconnected processes shaped by evolving meanings, role negotiations, and shifting family dynamics. These findings suggest that the DMT pathway extends beyond an informational and clinical trajectory to encompass ongoing emotional and relational processes requiring negotiation and recalibration.

## Theme 3: Systemic and collaborative support needs

This theme captures system-level support needs related to coordination and continuity of care across providers within the DMT care pathway. Although institutional arrangements are not directly observable to patients and informal caregivers, their accounts of confusion, uncertainty, and discontinuity reflect the experiential consequences of these structural conditions. Across interviews, participants described role ambiguity, inconsistent handover processes, limited availability of psychosocial support, and fragmented integration across the DMT care pathway.

### Subtheme 3.1 The need for clarity regarding primary care physicians’ roles in DMT eligibility assessment and post-diagnostic support

This subtheme captures inconsistencies and ambiguities in the role delineation between primary care physicians and DMT-designated institutions.

#### Code 3.1a Inconsistencies in DMT eligibility evaluations between primary care physicians and DMT-designated institutions

Primary data indicated that participants expressed uncertainty regarding why primary care physicians were unable to determine DMT eligibility accurately.

*My primary care physician recommended DMT*,* so I expected it to be a promising new treatment. I was shocked and disappointed to learn I wasn’t eligible. Why couldn’t my primary care physician evaluate properly? (Pt2)*

Secondary data highlighted structural factors underlying such discrepancies. Service providers noticed substantial variation among primary care physicians in their ability to assess very early-stage dementia.

*Some primary care physicians lack the skills needed to appropriately assess very early-stage dementia or mild cognitive impairment. (CS4)*

Others pointed out variation in primary care physicians’ recognition that these stages are now targets for DMT.

*There is also limited understanding that these conditions are now targets of treatment*,* and I think this is becoming a barrier to DMT implementation. (Dr1)*

Beyond differences in diagnostic capacity, providers also described uncertainty regarding how responsibilities for pre-referral screening and formal eligibility determination should be divided between primary care physicians and DMT-designated institutions.

*Before referring patients to DMT-designated institutions*,* I advise primary care physicians to confirm that there are no MRI contraindications or prior microbleeds*,* and to explain that DMT is administered by intravenous infusion. On the other hand*,* regarding the MMSE*,* scores can vary by a few points depending on the method and version used*,* so dementia staging should be assessed by trained staff at DMT-designated institutions. (Dr5)*

These narratives reflect a tension between maintaining appropriate screening standards and preventing under-referral, situated within ongoing negotiations over role delineation between primary care physicians and DMT-designated specialists. Some providers implied that over-triage within primary care may be more acceptable than the risk of excluding patients who might benefit from specialist evaluation.

#### Code 3.1b Ambiguity in post-diagnostic support roles between primary care physicians and DMT-designated facilities

Primary data indicated that participants expressed uncertainty about whom to consult for dementia-related concerns during DMT.

*I would consult my primary care physician about everything*,* because he knows me best. (Pt1*,* Pt7)*

*I don’t talk to my primary care physician about dementia because I’m already seeing a dementia specialist hospital for DMT. (Pt3)*

*My primary care physician is so busy that I hesitate to consult him. (IC6)*

*I’m not sure what I can talk about with the DMT doctor besides the infusion. (Pt5*,* Pt7)*

These narratives indicate substantial variation in how patients conceptualized the role of their primary care physicians within the DMT pathway, resulting in divergent expectations regarding responsibility for post-diagnostic care.

Secondary data similarly showed that service providers noted a lack of clear delineation of responsibilities between primary care physicians and DMT-designated institutions.

*After DMT starts*,* primary care physicians often leave dementia care*,* including post-diagnostic care*,* to the DMT facility*,* which makes me feel uncomfortable. (Dr6*,* SW4)*

This ambiguity was reported to result in situations in which neither side assumed responsibility for post-diagnostic support.

*For about eighteen months*,* patients receive care from both their primary care physician and the DMT-designated facility. But if they already have a primary care doctor*,* providing post-diagnostic support can create conflict*,* so we usually do not offer it. (Dr9)*

These findings indicate that ambiguity in role delineation contributed to discontinuity in post-diagnostic care.

Taken together, Subtheme 3.1 indicates that variability in DMT eligibility evaluation and post-diagnostic support arose from ongoing ambiguity in role delineation between primary care physicians and DMT-designated institutions. Primary narratives describe confusion and disappointment when expectations formed in primary care did not align with specialist assessments, as well as divergent understandings of whom to consult during treatment. Secondary perspectives highlight differences in diagnostic capacity, variation in recognition of early-stage dementia as a treatment target, and tensions regarding appropriate levels of pre-referral screening. Together, these findings suggest that challenges in both referral and post-diagnostic care may have been shaped not only by differences in clinical expertise, but also by evolving and sometimes unclear boundaries of responsibility within the DMT care pathway.

### Subtheme 3.2 The need for coordinated post-DMT handover and clearly assigned follow-up responsibility

This subtheme captures structural discontinuities in care transitions following DMT, particularly when eligibility is denied, DMT is discontinued or completed.

#### Code 3.2a Unclear post-DMT referral pathways

Primary data indicated that participants expressed uncertainty and discomfort regarding post-DMT transitions, including ineligibility, discontinuation, or treatment completion, despite being informed that the standard pathway involved returning to primary care physicians.

*When they told us that the DMT can’t be continued and that our visits to the clinic would also end*,* I felt as if we were being abandoned. (IC4)*

Some questioned whether non-specialists could adequately address dementia-related concerns.

*Where should we go after DMT ends? The physician told us that our primary care physician will take over*,* but he is not a specialist. (IC6)*

Secondary data showed that service providers observed that, although redirecting patients to their primary care physicians is standard practice after ineligibility or completion, coordination across institutions remains insufficient.

*When patients are deemed ineligible or complete DMT*,* they are usually redirected to their primary care physicians. But if they don’t have one*,* they are often not referred anywhere. (CP2)*

Providers also noted that the lack of systematic coordination often leaves patients confused.

*There is very little coordination across institutions*,* so patients often do not know where to go after DMT completion or discontinuation. (CS4)*

*I think the local government needs to establish a more formal referral pathway. This is difficult to address at the level of individual institution. (Dr5)*

These accounts illustrate how the standard referral pathway of returning to primary care physicians did not consistently translate into a sense of continuity or support, and in some cases contributed to feelings of abandonment and uncertainty during post-DMT transitions.

#### Code 3.2b Delay in accessing post-diagnostic support

While Subtheme 3.2a highlights patients’ and caregivers’ experiences of feeling unsupported or unsettled during post-DMT transitions, Subtheme 3.2b suggests that these experiences reflect a broader structural ambiguity not limited to transition points.

Secondary data described a lack of clearly designated responsibility for post-diagnostic support, including during the DMT period. This ambiguity was described as contributing to delays in accessing non-pharmacological and psychosocial interventions.

*I feel that because responsibility for post-diagnostic support is not clearly assigned*,* the system ends up prioritizing pharmacological treatment. As a result*,* non-pharmacological interventions often get delayed. (Dr2)*

These accounts illustrate how the absence of clearly assigned responsibility for post-diagnostic support contributed to delays in accessing non-pharmacological and psychosocial care.

Taken together, Subtheme 3.2 indicates that challenges in post-DMT care arose from both misalignment in referral transitions and ambiguity in follow-up responsibility. While a standard pathway of returning to primary care physicians was formally in place, it did not consistently provide patients and caregivers with a sense of continuity or security, particularly when specialist involvement ended. At the same time, secondary perspectives indicate that responsibility for post-diagnostic support was not consistently defined even during the DMT treatment period, resulting in delays in accessing non-pharmacological and psychosocial care. Together, these findings highlight how gaps in both transition alignment and role assignment shaped emotional experiences and the organization of support across the DMT trajectory.

### Subtheme 3.3 The need for multidisciplinary collaboration to provide post-diagnostic support

This subtheme reflects gaps in psychosocial care capacity within both primary care settings and DMT-designated institutions, highlighting the need for stronger multidisciplinary collaboration.

#### Code 3.3a Insufficient psychosocial care capacity across primary care and DMT-designated institutions

Primary data indicated that participants reported that access to post-diagnostic support was not systematically ensured within the DMT care pathway.

*It was just by chance that one of my brother’s colleagues introduced him to community dementia supporting services.(IC2)*

Some participants perceived that hospital-based services were focused primarily on medical procedures rather than broader psychosocial support.

*Hospital staff is so busy that I don’t think they can provide that kind of support. (IC2)*

Secondary data similarly showed that service providers reported insufficient availability of social workers, psychologists, and other professionals required for psychosocial care in both primary care settings and DMT-designated institutions.

*Primary care physicians usually do not hire social workers or psychologists for their clinics. It seems to me that it is unrealistic to expect that they would provide post-diagnostic support. (Dr3*,* Dr4)*

As a result, physicians were often the sole providers of post-diagnostic support, which was described as insufficient given the complexity of patients’ psychosocial needs.

*At DMT-designated institutions outside of MCDs*,* like ours*,* we don’t have social workers or psychologists available for DMT patients. So I end up managing all support needs myself*,* which is impossible. (Dr8)*

Notably, one physician described how the frequency of infusion visits created opportunities for relational engagement. In some cases, repeated encounters allowed psychosocial conversations to unfold within routine medical visits, suggesting that relational continuity may emerge incidentally from the treatment structure itself rather than from formally organized multidisciplinary support.

*We meet every two weeks*,* so we become close. We came to talk about their daily life and think together. Maybe it is the relationship*,* rather than the medication itself*,* that improves well-being. (Dr5).*

These accounts illustrate how limited psychosocial workforce capacity across care settings constrained systematic post-diagnostic support, while, in some cases, repeated clinical encounters created informal opportunities for relational engagement.

Taken together, Subtheme 3.3 indicates that psychosocial support within the DMT pathway was not systematically embedded in multidisciplinary structures across care settings. Primary narratives describe reliance on informal or chance-based pathways to community resources, while secondary perspectives highlight workforce shortages and infrastructure limitations. In the context of limited psychosocial workforce capacity, physicians frequently assumed the role of providing psychosocial support within routine DMT encounters. Although repeated clinical visits may foster relational engagement, such support appears to emerge primarily through individual clinical practice and relational dynamics rather than through structurally embedded multidisciplinary systems.

### Subtheme 3.4 Toward an integrated dementia care system that includes DMT

This subtheme is informed primarily by professional perspectives because mechanisms of institutional integration are not directly accessible to patients and caregivers. While primary narratives illuminate the lived experience of fragmentation and uncertainty (as described in Subthemes 3.1–3.2), professional accounts provide insight into the structural arrangements that generate those experiences. In this sense, Subtheme 3.4 does not introduce a separate phenomenon but rather explicates the systemic conditions underlying earlier patient-reported disruptions.

#### Code 3.4a Fragmented linkage between DMT-designated institutions and MCDs

Secondary data indicated fragmentation between DMT-designated institutions and Medical Centers for Dementia.

*Post-diagnostic support is usually provided within MCDs and psychiatric services*,* while DMT is mainly delivered by neurologists and neurosurgeons. Sometimes they don’t even know what post-diagnostic support involves. (PSW5)*

Others emphasized that clearer coordination mechanisms were needed.

*I think effective collaboration between DMT-designated institutions and MCDs is ideal for providing post-diagnostic support*,* including support after DMT discontinuation and for those deemed ineligible. If that kind of collaboration existed*,* patients could continue receiving appropriate medical and social support as dementia progresses. (Dr4)*

These accounts illustrate how parallel service structures—neurology-led DMT provision and psychiatry- or MCD-led post-diagnostic support—operate with limited integration, resulting in fragmented care transitions.

#### Code 3.4b DMT being positioned too narrowly within the broader continuum of dementia care

Beyond structural separation between service domains described in Code 3.4a, providers also highlighted how DMT was symbolically positioned within dementia care discourse. Secondary data suggest concern that DMT may be framed as an exceptional or superior intervention, potentially narrowing perceptions of legitimate treatment options within the broader care continuum.

*If patients view anything other than DMT as an “inferior treatment*,*” they may be reluctant to consider other therapies or non-pharmacological approaches. I think we need to send a more positive message that options beyond DMT are also valuable. (Dr1)*

Furthermore, excessive emphasis on DMT was described as potentially marginalizing patients from the broader continuum of dementia support.

*If we place too much emphasis on DMT*,* we may end up in a situation where those who seek it become marginalized from the existing dementia care system. (PSW5)*

These accounts illustrate how the symbolic prominence of DMT within dementia care discourse may narrow perceptions of legitimate treatment options and unintentionally marginalize other forms of support.

Taken together, Subtheme 3.4 indicates that institutional segmentation—both structural and symbolic—shaped continuity within the DMT care pathway. Professional accounts describe limited integration between parallel service systems, as well as the positioning of DMT as a distinct or exceptional intervention within dementia care. While patients and caregivers experience this segmentation as uncertainty or discontinuity (as described in Subtheme 3.1–3.2), provider perspectives clarify the systemic configurations that underlie those experiences. Together, these findings suggest that integration challenges arise not from the absence of services, but from the ways in which DMT is organizationally and discursively situated within the broader dementia care continuum.

Across Subthemes 3.1–3.4, a consistent pattern emerges: systemic role ambiguity and institutional segmentation appear to shape the DMT care pathway. Primary narratives describe uncertainty and misaligned expectations during referral and treatment processes, as well as feelings of being unsupported at post-DMT transition points. Secondary perspectives highlight variability in role delineation, insufficient coordination of handover processes, uneven psychosocial workforce capacity, and limited integration between parallel dementia-care systems. Together, these findings suggest that challenges in DMT implementation are closely linked to structural boundary issues and coordination dynamics within the broader dementia care system.

## Discussion

### Overview of findings from patients’ and informal caregivers’ narratives

From the perspectives of patients and informal caregivers, the introduction of DMTs was experienced not merely as access to a new therapeutic option but as a process that reshaped expectations, relationships, and care responsibilities over time. Rather than unfolding as a linear clinical pathway, the DMT trajectory was characterized by shifting understandings, emotional negotiation, and evolving role configurations across assessment, treatment, and post-DMT phases.

When these lived experiences are considered alongside professional perspectives, broader structural tensions become visible. Ambiguities in role delineation, variability in referral and follow-up processes, and uneven psychosocial capacity influenced how patients and caregivers navigated the DMT pathway. In this sense, DMT functioned less as a discrete therapeutic innovation and more as a lens through which existing discontinuities within post-diagnostic dementia support became visible.

Together, these findings suggest that effective DMT implementation requires more than technical readiness for drug delivery. It also depends on sustained informational clarity, relational continuity, and coordinated post-diagnostic support embedded within the broader dementia care continuum. By centering patient and caregiver experiences, this study extends current debates beyond eligibility and consent to consider how pharmacological innovation reshapes the relational and systemic organization of dementia care.

### Informational support needs: continuity, accessibility, and service responsibility

The findings suggest that informational challenges in the DMT era are not confined to initial access to information, but are embedded in how health services structure continuity, reinforcement, and responsibility for information provision across the care pathway. While access to information at the onset varied, patients and caregivers often described navigated informational environments shaped by uneven distribution of reliable sources and exposure to public discourse that could amplify uncertainty.

Although some participants demonstrated adequate understanding, sustaining and updating understanding over time emerged as a central challenge. Even when explanations were initially clear, difficulties arose in recalling, contextualizing, or reinterpreting information as treatment progressed or circumstances changed. This instability may not be fully explained by cognitive factors alone; it also reflects service structures that offer limited opportunities for iterative dialogue, confirmation, and recalibration of expectations.

Informational discontinuity became particularly salient at transition points within the DMT pathway, including ineligibility, treatment discontinuation, or completion, where ambiguities in follow-up responsibility amplified uncertainty. These findings align with Belder et al. [1], who emphasize that careful communication and expectation management are essential, given that many patients will not meet eligibility criteria and will require alternative care pathways.

Taken together, informational support in the DMT era should be conceptualized as a longitudinal service function embedded within care pathways. Rather than being limited to one-time disclosure at the point of consent, it requires clearly assigned responsibility for ongoing communication, reinforcement, and adjustment over time.

### Emotional and psychosocial support needs: navigating stigma, family tension, and meaning reconstruction

The findings indicate that emotional and psychosocial challenges in the DMT process appears to be shaped not only by diagnosis or treatment itself, but also by how clinical encounters are structured over time. Experiences of stigma, emotional ambivalence, and family-related tension were influenced by how information was communicated, how expectations were managed, and when opportunities for dialogue were provided.

Stigma—both public and internalized—played an ambivalent role in treatment-seeking and decision-making. In some cases, it motivated pursuit of DMT in hopes of avoiding feared futures; in others, it intensified anxiety and complicated diagnosis acceptance. These patterns suggest the importance of acknowledging and addressing emotional responses early in the care pathway, rather than assuming that psychosocial support can be deferred until later disease stages.

Family dynamics represented another psychosocial dimension influenced by service organization. The way DMT pathways rely on family involvement often generated emotional and practical strain, particularly when expectations regarding treatment benefits diverged. Limited opportunities for facilitated dialogue within clinical settings appears to contributed to unspoken tensions and misaligned expectations, suggesting that psychosocial support may need to be more consistently embedded within routine service pathways rather than treated as an optional component of DMT delivery.

Uncertainty regarding treatment effects further contributed to emotional strain among patients and caregivers. When benefits were difficult to perceive, anxiety, discouragement, and ambivalence about treatment continuation were common. Over time, some families recalibrated expectations and developed more sustainable interpretations of treatment, underscoring that psychosocial adaptation is a dynamic process requiring ongoing support rather than one-off interventions.

For individuals deemed ineligible for DMT or who discontinued treatment, psychosocial needs became particularly salient during transition periods. Initial disappointment was often followed by gradual engagement with non-pharmacological and community-based support, pointing to processes of meaning reconstruction beyond pharmacological options.

### Systemic and collaborative support needs: from fragmented responsibilities to integrated care

From a system-level perspective, the findings highlight how ambiguities in roles and responsibilities across the DMT care pathway may create challenges for coordination and continuity of care. Unclear delineation of responsibilities for eligibility assessment, and post-diagnostic follow-up, combined with variability in referral pathways and assessment expertise, contributed to inconsistent patient experiences and, at times, frustration.

Transitions following ineligibility, treatment discontinuation, or completion appear to represent particularly sensitive junctures. Although returning to primary care was described as the standard approach, handovers were not always experienced as seamless, reflecting systemic gaps in coordination that were sometimes associated with delayed access to psychosocial and non-pharmacological support. Service providers further highlighted limited psychosocial care capacity across both primary care and DMT-designated institutions. Restricted availability of multidisciplinary professionals constrained the ability to address complex support needs, often leaving physicians to manage issues extending beyond biomedical treatment— an essential component of comprehensive dementia care that may be difficult to sustain without adequate multidisciplinary support. Together, these patterns underscore the importance of clearer integration between DMT delivery pathways and existing dementia-care infrastructures, particularly MCDs, which were originally designed to coordinate multidisciplinary post-diagnostic support.

These findings clarify how structural discontinuities in post-diagnostic support emerge in practice, deepening earlier observations in the Japanese context [12]. They also align with concerns raised by Cooper et al. [14] that, prioritizing DMT implementation without appropriate investment may risk diverting resources from the larger group of persons living with dementia who are ineligible for these therapies. Persistent structural ambiguity also raises questions regarding accountability and fairness in access to post-diagnostic support within evolving models of dementia care.

### Ethical and practical implications for health service design

The findings of this study suggest that challenges emerging in the DMT era are unlikely to be resolved through individual communication efforts alone, as they are closely intertwined with health service design and organizational arrangements. Questions of equity in informational access, continuity of support, and allocation of responsibility for decision-making extend beyond clinician–patient interactions to the configuration of care pathways and systems.

From a health services perspective, these challenges carry important ethical and practical implications. The longitudinal and relational nature of decision-making in dementia care calls for service systems that facilitate ongoing dialogue, clarify roles delineation across providers, and distribute responsibility more transparently and equitably across institutions. Structural alignment can promote clearer institutional accountability, allowing patients and families to exercise agency in care decisions without compensating for structural gaps in coordination.

### Broader implications for dementia care

Although this study was conducted in Japan, its findings may unfold relevance for other healthcare systems preparing for or implementing DMTs. Japan’s early adoption of DMTs illustrates how pharmacological innovation may, at times, advance more rapidly than the parallel development of psychosocial and organizational infrastructures, thereby revealing underlining vulnerabilities in existing dementia-care systems. Integrating DMTs within comprehensive dementia-care pathways may enable other countries anticipate similar challenges and design services that promote continuity, equity, and relational care.

Japan’s nationally coordinated dementia-care framework provides a distinctive context in which to observe how pharmacological innovation interacts with an already structured system. It is important to acknowledge that the interviews were conducted during the first year of DMT implementation in Japan, when service pathways were still evolving. Participants’ narratives therefore tended to foreground uncertainty and unmet needs rather than fully developed examples of mature practice. Moreover, the study’s focus on support needs may have further emphasized challenges relative to successfully resolved experiences.

While this temporal positioning limits the identification of best-practice models, it also captures a formative phase of service adaptation that other healthcare systems may encounter during early DMT adoption. The findings offer insight into structural and relational vulnerabilities that can arise before coordination mechanisms and multidisciplinary frameworks are fully embedded. At the same time, emerging examples within the data—such as relational continuity during infusion visits, community-based engagement following ineligibility, and growing recognition of the need for clearer role delineation—suggest that good practice in the DMT era may depend less on expanding pharmacological capacity alone and more on embedding structured communication, relational continuity, and integrated psychosocial support within care pathways from the outset.

### Strength, limitations, and future directions

This study offers qualitative insight into the psychosocial and systemic implications of early DMT implementation within real-world dementia care. By integrating perspectives from patients, informal caregivers, clinicians, and community-based professionals, it illuminates the multi-layered support needs that accompany the introduction of new pharmacological therapies. While patient and caregiver participants were recruited primarily from metropolitan institutions, the inclusion of clinicians working across diverse care settings strengthened understanding of broader system-level dynamics. Professional perspectives were used to contextualize, rather than directly compare stakeholder groups, supporting analytic coherence across heterogeneous data sources.

Several limitations should be noted. The study may underrepresent individuals with the greatest unmet needs—such as those with limited social support or substantial caregiving strain—who may face barriers to accessing DMT pathways altogether. Furthermore, the number of participants, particularly people living with dementia and informal caregivers, was modest, and secondary participants were drawn from multiple professional categories, resulting in small subgroup sizes. Finally, ethical and psychosocial issues related to Apolipoprotein E (APOE) genotyping could not be examined, as none of the participants underwent genetic testing during evaluation. Given the ethical complexity of genetic risk disclosure, future research should explore how APOE testing may influence understanding, decision-making, and emotional responses.

Nevertheless, insights from Japan—where DMTs have been introduced within a nationally coordinated dementia-care framework—offer valuable lessons regarding the ethical, psychosocial, and organizational challenges of early DMT adoption. Future research should examine longitudinal adaptation among patients and caregivers, cross-national experiences, and ethical implications of DMT access across different policy environments. It will also be important to further explore how DMT-related experiences and support needs may vary across rural and urban settings and according to gender, educational background, comorbidities, and other dimensions of diversity.

## Conclusion

This study demonstrates, from the perspectives of patients and informal caregivers, that the introduction of DMTs has brought longstanding gaps in dementia care systems into sharper focus across informational, psychosocial, and systemic domains. Addressing these challenges requires aligning pharmacological innovation with health service design that ensures continuous information provision, integrated psychosocial care, and coordinated follow-up across care pathways.

Because DMT involves sustained engagement from patients and informal caregivers, it highlights the importance of service structures that provide accessible and comprehensible information, accommodate fluctuating understanding over time, and support ongoing family involvement. These findings suggest that autonomy in dementia care is enacted through evolving relational processes among patients, informal caregivers, and clinicians, grounded in ongoing dialogue and shared understanding over time.

At a systems level, the introduction of DMT underscores the need to integrate pharmacological advances with psychosocial resources and referral processes that are not only formally defined but experienced as continuous and supportive. It also calls for clearly delineated roles accompanied by active and coordinated collaboration among primary care, specialized institutions, and MCDs. When such integration is embedded within care systems, therapeutic innovation can be accompanied by continuity, equity, and relational trust. Ultimately, the value of DMT will depend not only on pharmacological efficacy but on the capacity of health systems to situate these therapies within care pathways that uphold fairness, sustain trust, and support the relational realities of living with dementia.

## Supplementary Information

Below is the link to the electronic supplementary material.

Supplementary Material 1

## Data Availability

The qualitative datasets generated and analyzed during the current study are not publicly available due to the sensitive nature of the interview data and the risk of participant identification. Anonymized data may be made available from the corresponding author on reasonable request, subject to approval by the relevant ethics committee and data use agreements.

## Abbreviations

AD: Alzheimer’s Disease
DMT: Disease-modifying therapy
MCD: Medical Centers for Dementia
