# Supplementary material for: Reframing dementia care in the era of disease-modifying therapies: informational, psychosocial, and systemic insights from Japan
Source: BMC Health Serv Res. 2026 Mar 30;26:660. doi: 10.1186/s12913-026-14472-8 (PMC13154527; doi:10.1186/s12913-026-14472-8)
Supplement: Supplementary file 1 — Supplementary Material 1 [file 12913_2026_14472_MOESM1_ESM.docx]

**Supplementary File 1: Interview Guides**

**A. Interview Guide for Patients and Informal caregivers**

**1. Participant’s Characteristics**

Please provide the following information: age group and sex.

Please also share details about your usual daily life and caregiving situation.

**2. Psychosocial Needs Emerging with the Introduction of Disease-Modifying Therapies (DMTs) for Dementia**

**2a. DMT Eligibility**

-How did you feel when you received a diagnosis of dementia/mild cognitive impairment and underwent evaluation for DMT?

Please share the reasons for your feelings.

-After being informed that you were eligible/ineligible for treatment, did you experience any emotional changes?

If yes, what kind of changes occurred?

-Through the experience (regardless of eligibility), did you notice any changes in your daily life? If so, what kind of changes?

- Through this experience (regardless of eligibility), have there been any changes in your relationship with your family member(s)?

If yes, what kind of changes?

**2b. Conflicts Between Patients, Informal caregivers, and Within Families**

-What initially prompted you to consider DMT as a possible option?

-Who initiated the decision to seek medical evaluation for DMT?
-What were your feelings or thoughts regarding DMT? (e.g., anxiety, hesitation, hope, etc.)

**2c. Perceived Treatment Effects**

For participants who received DMT

-Since beginning DMT, have you observed any changes that made you feel the treatment was having positive or negative effects, including perceived effectiveness?

-How, if at all, have these perceptions influenced your daily life or emotional state?

**2d. Care Coordination**

-During the DMT process, to whom did you feel comfortable talking when you had concerns?

**3. Overall opinion**

-Reflecting on the entire process so far (from evaluation to today) what kinds of support would have been most helpful for you?

**B. Interview Guide for Professionals**

**1. Basic Information**

Please provide the following details:

Sex, profession, years of experience, and your role in delivering disease-modifying therapy (DMT).

**2. Understanding of Explanations of DMT Effects, Limitations, and Side Effects**

- To what extent and how do patients appear to understand these explanations generally?
- To what extent and how do informal caregivers appear to understand them generally?
- What aspects of the explanation appear to be difficult to understand or are frequently misunderstood?

**3. Acceptance of the Diagnosis**

- How do patients typically understand and come to terms with the diagnosis?
- How do informal caregivers typically understand and come to terms with the diagnosis?
- Which aspects of the explanation of diagnosis appear to be difficult to understand or are commonly misunderstood?

**4. Understanding and Acceptance of APOE Genetic Test Results**

For patients who underwent APOE genetic testing and their caregivers (if applicable)
- How do patients typically appear to understand and accept their results?
- How do family members typically appear to understand and accept them?
- What aspects of the explanation appear to be difficult to understand or are commonly misunderstood?

**5. Understanding and Acceptance of Ineligibility for DMT**

For patients and informal caregivers determined to be ineligible for DMT

- What kinds of psychosocial support needs do you perceive among these patients and informal caregivers?

- After referral back to their primary care physician, do you think they actually receive post-diagnostic support for dementia?

- Do you make any special arrangements or handovers to ensure that post-diagnostic support is provided?

**6. Conflicts Between Patients, Informal caregivers, and Within Families**

- In your experience, how was the patient’s own intention reflected in the decision-making process? Can you recall any cases that illustrate this?

- Please describe any cases in which you felt the patient’s opinion was respected.

- Please describe any cases in which you felt the patient’s opinion was not adequately respected.

- Among patients who personally requested DMT and those whose informal caregivers were more enthusiastic about treatment, what psychosocial support needs do you recognize in relation to (1) awareness of the current condition, (2) attitudes toward treatment, or (3) attitudes toward the strain of hospital visits?

**7. Perceptions of Treatment Effects**

- How do patients and informal caregivers generally perceive the treatment effect?

- What psychosocial support needs are apparent at that stage?

**8. Barriers to Accessing DMT**

-What physical or psychological barriers do patients and informal caregivers perceive regarding DMT itself?

-What physical or psychological barriers do they perceive in accessing the DMT-designated institutions?

**9. Coordination of Care**

-What types of collaboration do you have with DMT-designated institutions/primary care physicians/Medical Centers for Dementia?

-In what situations do you feel this collaboration helps address patients’ psychosocial support needs?

- In your opinion, at what stage, by whom, and in what form should post-diagnostic support ideally be provided?

- Do you think such support is currently being provided?

If yes, at what stage and in what form is it actually provided?
